# The Impact of Postoperative Albumin Levels on Furosemide Efficacy in Infants with Congenital Heart Disease

**DOI:** 10.3390/life14121679

**Published:** 2024-12-18

**Authors:** Ayşegül Aşır, Bedri Aldudak, Nilüfer Matur Okur

**Affiliations:** 1 Clinics of Pediatrics, Gazi Yaşargil Training and Research Hospital, 21090 Diyarbakır, Turkey; 2 Clinics of Pediatric Cardiology, Gazi Yaşargil Training and Research Hospital, 21090 Diyarbakır, Turkey; 3 Clinics of Neonatology, Gazi Yaşargil Training and Research Hospital, 21090 Diyarbakır, Turkey

**Keywords:** albumin, furosemide, congenital heart disease, cardiopulmonary bypass

## Abstract

Postoperative fluid overload is associated with increased mortality and morbidity in infants with congenital heart disease (CHD). Loop diuretics, such as furosemide, are commonly used to prevent fluid overload in the postoperative period. This study aimed to investigate the effect of postoperative albumin levels on the efficacy of furosemide after surgery in infants with CHD. From 1 January 2017 to 31 December 2022, postoperative albumin levels, total furosemide doses, and three-day postoperative diuresis levels were retrospectively analyzed in 186 patients aged 0–1 years who underwent cardiopulmonary bypass at the Pediatric Intensive Care Unit, Diyarbakır Gazi Yaşargil Training and Research Hospital. Demographic and clinical parameters, along with urine output in the first 6 h, first 24 h, 24–48 h, and 48–72 h postoperatively, were recorded. Patients were divided into two groups based on their albumin levels: normal albumin (≥30 g/dL) and hypoalbuminemia (<30 g/dL). A common protein interaction network for albumin and furosemide was constructed using Cytoscape software (version 3.10.2). Of the 186 patients, 79 (42.5%) were male and 107 (57.5%) were female, with a median age of 97.5 days (range 1–360 days). Furosemide doses were higher in hypoalbuminemic patients on postoperative days 1 and 2 compared to normoalbuminemic patients. On postoperative day 1, hypoalbuminemia was more prevalent in patients with oliguria, whereas normoalbuminemia was significantly higher in patients with normouria and polyuria. Furosemide doses were significantly higher in patients with oliguria than in those with normouria in the first 6 h and lower in patients with polyuria compared to those with normouria. A positive correlation was observed between albumin levels and furosemide efficacy on postoperative day 2. Additionally, a positive correlation existed between albumin levels on postoperative day 1 and urine output in the first 6 and 24 h postoperatively. Furosemide efficacy and urine output were positively correlated in the postoperative period. Mortality risk was significantly higher in hypoalbuminemic patients on postoperative days 1 and 2, as well as in patients with oliguria in the first 6 and 24 h postoperatively. Network analysis revealed that albumin was directly involved in furosemide’s target network, along with six other proteins within the common interaction network. Diuresis levels were significantly lower in hypoalbuminemic patients. We suggest that the effectiveness of furosemide is reduced because it cannot bind to albumin at sufficient levels. The effective management of albumin levels may enhance furosemide efficacy and improve postoperative outcomes in infants with CHD.

## 1. Introduction

Pediatric cardiac surgery is a crucial intervention for congenital heart disease (CHD), a leading cause of morbidity and mortality in children [1]. Despite advancements in surgical techniques and perioperative care, managing postoperative complications remains a significant challenge [2,3]. One of the primary concerns is fluid overload, which can lead to acute kidney injury (AKI), prolonged mechanical ventilation, and extended intensive care unit (ICU) stays [4,5]. Effective fluid management is critical to improving outcomes in these patients.

Albumin, a major plasma protein, plays a vital role in maintaining oncotic pressure and vascular integrity [6]. Hypoalbuminemia, or low serum albumin levels, is frequently observed in critically ill patients and has been associated with poor prognosis, increased morbidity, and mortality [7]. In pediatric patients undergoing cardiac surgery, hypoalbuminemia can result from a combination of factors, including surgical stress, inflammatory responses, and capillary leak syndrome post-cardiopulmonary bypass (CPB) [8].

Several studies have highlighted the prognostic significance of serum albumin levels in various clinical settings. For instance, low preoperative albumin levels have been linked to adverse outcomes, including increased risk of infections, longer hospital stays, and higher mortality rates [9]. Similarly, postoperative decreases in albumin levels have been correlated with complications such as AKI and prolonged mechanical ventilation [10]. These findings underscore the importance of monitoring and managing serum albumin levels in pediatric cardiac surgery patients.

Furosemide, a commonly used loop diuretic, is often administered to manage fluid overload in postoperative pediatric cardiac patients [11]. Furosemide is associated with numerous adverse effects across various organ systems. In the gastrointestinal system, it can cause pancreatitis, elevated hepatic enzymes, diarrhea, constipation, nausea, and vomiting. Allergic reactions include anaphylactic responses, systemic vasculitis, and interstitial nephritis. Neurologic side effects may manifest as headaches, blurred vision, and vertigo [12,13,14]. Hematologic complications are noted with occurrences of purpura, rash, pruritus, and urticaria. Cardiovascular effects include orthostatic hypotension, elevated cholesterol, and increased triglycerides. Metabolic disturbances associated with furosemide involve hyperglycemia, hyperuricemia, hypokalemia, and hypomagnesemia. Overdose with furosemide presents primarily as hypovolemia, dehydration, electrolyte disturbances, hypotension, hypochloremic alkalosis, and hypokalemia. The management of furosemide toxicity is supportive, primarily focused on the replenishment of fluids and electrolytes to mitigate deficits. The regular assessment of serum electrolyte levels, arterial blood gas analysis, and blood pressure is crucial in these cases [13,14,15,16].

However, its efficacy can be influenced by the patient’s albumin levels. Since furosemide binds to albumin for transport to its site of action in the kidneys, hypoalbuminemia can impair its effectiveness, necessitating higher doses to achieve the desired diuretic response [17,18]. This interaction highlights the need for a comprehensive approach to fluid management that considers both diuretic administration and albumin supplementation.

In this study, we aim to investigate the relationship between serum albumin levels, furosemide efficacy, and urine output in pediatric patients undergoing cardiac surgery for CHD. By analyzing preoperative and postoperative albumin levels, urine output, and furosemide doses, we seek to provide insights into optimal fluid management strategies that can improve postoperative outcomes in this vulnerable patient population.

## 2. Patients and Methods

### 2.1. Study Design

This study included infants with CHD who were operated on using CPB between 1 January 2017 and 31 December 2022. This study was a retrospective design cohort study. Demographic data of the patients included in this study, including age, gender, type of birth, place of birth, birth weight, and the weight at which they arrived at the hospital, were recorded. Preoperative serum albumin levels of the patients and postoperative albumin levels and urine output levels at the 24th, 48th and 72nd hours were recorded. Albumin values above 30 g/dL were considered normal, and these patients were grouped as normoalbuminemic (Group 1) and hypoalbuminemic (Group 2) according to this criterion. Urine output levels of the patients were calculated as mL/kg/h. Diuresis levels were classified as oliguria (urine output is less than 1 mL/kg/h for infants and young children), normuria (urine output is generally between 1 and 2 mL/kg/h for infants and young children), and polyuria (urine output exceeds 3 mL/kg/h in infants and young children) [19].

The amount of intravenous furosemide received by the patients was recorded in mg/kg/day. Within the scope of clinical features, congenital heart disease diagnosis, vasoactive inotropic drugs and fluids administered, duration of respiratory support, preoperative and postoperative urine output, laboratory parameters, cardiopulmonary bypass duration, aortic clamping duration, and mortality data were recorded. Patients who died within the first 48 h postoperatively, those with a known kidney disease, hydrops fetalis, or severe systemic infections (e.g., sepsis) prior to surgery, those with the presence of genetic syndromes, patients administered other diuretics or medications affecting renal function and fluid balance prior to or during the study period, those with severe electrolyte imbalances, and patients with incomplete data were excluded from this study. In our health center, albumin administration was a choice left to physicians.

### 2.2. Network Analysis of Albumin and Furosemide

To explore the molecular interactions that may support the relationship between albumin and furosemide, we conducted a protein–protein interaction (PPI) network analysis. This analysis was performed using Cytoscape software (version 3.10.2), integrating data from the STRING (Search Tool for the Retrieval of Interacting Genes/Proteins) and STITCH (Search Tool for Interactions of Chemicals) databases. Initially, individual PPI networks for albumin and furosemide were created. In STRING, albumin was queried to generate a network of up to 100 interactors. Similarly, STITCH was used to build a network of chemical–protein interactions around furosemide, also with a maximum of 100 interactors. A minimum confidence score of 0.4 was applied in both databases to filter interactions with moderate to high reliability, balancing the trade-off between interaction inclusivity and data accuracy [20]. To analyze the molecular pathways in which the common protein interactors participate, Kyoto Encyclopedia of Genes and Genomes (KEGG) pathway annotation was performed using the ShinyGO platform (version 0.81) [21]. Pathways with a false discovery rate (FDR) of less than 0.05 were considered statistically significant, and the top 10 significant pathways were ranked based on fold enrichment values.

### 2.3. Statistical Analysis

Statistical analyses in this study were performed using IBM SPSS Statistics for Windows, Version 26.0., and the limit of statistical significance was accepted as *p* < 0.05. A Mann–Whitney U test and Student’s *t* test were used to compare variables in two independent groups, and chi-square and Fisher’s exact tests were used to examine the relationship or differences between groups in terms of categorical variables. Relationships between continuous quantitative variables were summarized with the non-parametric Spearman correlation coefficient and associated *p* values. Friedman and repeated-measures ANOVA and Kruskal–Wallis models were used to examine the changes in repeated measurements over time, and the results of multiple comparison tests were given to determine the groups that created the difference.

## 3. Results

### 3.1. Features of Patients

The demographic and clinical characteristics of the patients are shown in Table 1. This table presents the basic demographic and clinical characteristics of the patients included in this study. It includes essential information such as age, gender, body weight, and key surgical durations. For instance, postoperative pulmonary hypertension was observed in 36.5% of patients, highlighting the significance of preoperative and intraoperative factors affecting postoperative outcomes. This high incidence underscores the importance of monitoring and managing these patients carefully during and after surgery.

### 3.2. Frequency of CDH Types

Table 2 shows the distribution of various congenital heart diseases (CHD) among the patients. The most common CHDs were an atrial septal defect combined with a ventricular septal defect (28.5%) and hypoplastic left heart syndrome (9.6%). Understanding the prevalence of different CHDs in the study population helps in tailoring specific perioperative and postoperative care strategies to improve outcomes for each CHD type.

### 3.3. Urine Output of Patietns

Table 3 categorizes patients’ urine output postoperatively, indicating oliguria, normal urea, and polyuria at different time intervals. For example, 53.7% of patients had polyuria in the first 6 h postoperatively. Tracking urine output is crucial as it is a significant indicator of renal function and fluid balance in postoperative care. Variations in urine output can inform the need for interventions to prevent complications such as renal failure.

### 3.4. Urine Output—Albumin Relationship

Table 4 compares urine output between patients who received albumin treatment and those who did not. While no significant difference was found in the first 6 h (*p* = 0.077), a significant difference was noted in the 24–48 h period (*p* = 0.02). Albumin therapy appears to influence urine output positively in the critical early postoperative phase, suggesting its role in fluid management and possibly improving renal perfusion and function.

### 3.5. Assocation of Albumin with Furosemide and Urine Output

Table 5 analyzes the correlation between albumin levels, furosemide use, and urine output. Significant positive correlations were observed between albumin levels on the first postoperative day and urine output at 6 and 24 h (*p* = 0.024 and *p* = 0.012). This indicates that maintaining albumin levels may enhance diuretic efficacy and urine output, emphasizing the importance of albumin in fluid management and renal function.

### 3.6. Albumin Effects on Furosemide and Urine Output

Regression analysis in Table 6 shows that albumin significantly affects both furosemide and urine output in the first 24 h postoperatively. Higher albumin levels correlated with increased urine output (*p* = 0.003), reinforcing the role of albumin in enhancing diuretic response and fluid balance, which is critical for preventing complications like fluid overload and renal dysfunction.

### 3.7. Effects of Albumin on Mortality and Urine Output

Table 7 presents the impact of postoperative albumin levels and urine output on mortality risk. Hypoalbuminemia on the first and second postoperative days significantly increased mortality risk (*p* < 0.001 and *p* = 0.008). Additionally, oliguria in the first 6 and 24 h postoperatively was associated with increased mortality risk. These findings highlight the prognostic importance of early postoperative albumin levels and urine output in predicting patient outcomes and guiding clinical interventions to reduce mortality.

### 3.8. Echocardiographical Parameters of Patients

Table 8 shows echocardiographic parameters, including EF, in hypoalbuminemic and normoalbuminemic groups. Echocardiographic parameters, including the ejection fraction (EF), were analyzed to assess cardiac function across the hypoalbuminemic and normoalbuminemic groups. The EF was significantly lower in the hypoalbuminemic group (mean ± SD: 55 ± 10%) compared to the normoalbuminemic group (60 ± 8%, *p* < 0.05), indicating impaired cardiac performance in patients with reduced albumin levels. Similarly, left ventricular (LV) end-diastolic and end-systolic diameters were higher in the hypoalbuminemic group, reflecting potential ventricular remodeling or dysfunction. These findings align with the hypothesis that hypoalbuminemia negatively impacts cardiac function, likely due to the systemic effects of inflammation, fluid overload, and impaired protein metabolism observed in these patients.

### 3.9. Molecular Analysis of Albumin-Furosemid Interaction

**Figure 1:**
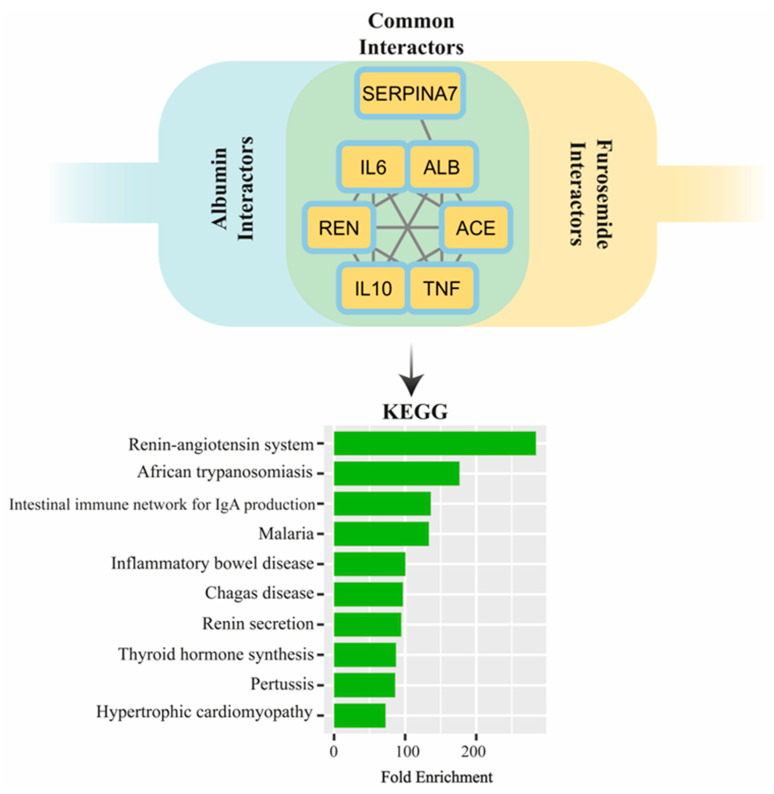
Common protein interactors between albumin and furosemide identified through PPI network analysis. The Venn diagram highlights shared interactors, including SERPINA7, IL6, ALB, REN, ACE, IL10, and TNF. Below, the KEGG pathway enrichment analysis of these common interactors is shown, with pathways ranked by fold enrichment.

Figure 1 illustrates the protein–protein interaction network involving albumin and furosemide. In the network analysis, among the proteins, albumin (ALB) was found to directly interact with furosemide. Additionally, six other proteins within the common interaction network were identified: angiotensin-converting enzyme (ACE), interleukin-10 (IL10), tumor necrosis factor (TNF), interleukin-6 (IL6), thyroxine-binding globulin (SERPINA7), and renin (REN). KEGG pathway enrichment analysis of these common interactors revealed that the most significantly enriched pathway was the renin–angiotensin system. Additional enriched pathways included the intestinal immune network for IgA production, inflammatory bowel disease, renin secretion, thyroid hormone synthesis, pertussis, and hypertrophic cardiomyopathy.

## 4. Discussion

Postoperative pediatric cardiac surgery is a highly complex process requiring vigilant monitoring and proactive management to prevent various complications. Intervention strategies aim to mitigate low cardiac output and prevent adverse sequelae in major organ systems [22]. Our study highlights the critical importance of managing capillary leak syndrome post-cardiopulmonary bypass (CPB) and addressing fluid overload due to high fluid administration levels. Postoperative fluid overload in patients with congenital heart disease (CHD) has been linked to acute kidney injury (AKI), prolonged mechanical ventilation, respiratory function deterioration, and extended ICU stays [23]. Monitoring urine output serves as a practical and immediate approach to assessing dose response to a single drug [24].

Numerous studies have highlighted that low albumin levels can indicate poor prognosis and malnutrition [25,26]. Our findings are consistent with previous research demonstrating that hypoalbuminemia is associated with increased morbidity and mortality among critically ill pediatric patients with CHD. Low serum albumin concentrations (<30 g/dL) were linked with higher mortality rates, postoperative infections, and longer hospital stays [10]. Henry et al. [27] and Kapoor et al. [28] reported significant decreases in albumin levels post-cardiac surgery and their correlation with mortality risk. Our study observed similar trends, with low postoperative albumin levels correlating with increased mortality, particularly in cyanotic patients where CPB-induced inflammatory responses are significant.

An observational study examined the relationship between preoperative and postoperative day 2 albumin levels, vaso-inotropic score (VIS), and ICU length of stay in infants undergoing CHD surgery. The negative correlation between albumin levels and both VIS and ICU stays reinforces the importance of preoperative and early postoperative albumin levels as predictive parameters for complicated and prolonged postoperative follow-ups [29].

CPB contributes significantly to fluid overload post-heart surgery, particularly in younger children, those with cyanotic heart lesions, and those undergoing complex surgeries [30]. Fluid overload is managed routinely with diuretics, with furosemide being the most commonly used loop diuretic in pediatric CHD patients [31,32]. Research indicates that children with cyanotic CHDs, which result in low oxygen levels, often experience impaired growth and development. This malnutrition can adversely affect recovery after cardiac surgery, leading to longer hospital stays and increased rates of infection [33]. Neonates undergoing procedures like the Norwood operation for hypoplastic left heart syndrome face higher risks of complications such as low cardiac output syndrome and arrhythmias, which can impact survival rates [34]. Patients with tetralogy of Fallot may experience arrhythmias and right ventricular dysfunction after repair, affecting long-term health [35]. Our study aligns with Ricci et al. [23], who found furosemide to be reliable in managing renal functions postoperatively. However, the effectiveness of diuretics can be compromised in hypoalbuminemic patients, as reduced albumin levels decrease the formation of the furosemide–albumin complex, limiting the drug’s transport to its site of action.

We observed a significant relationship between albumin levels and urine output within the first 24 h postoperatively. Higher doses of furosemide were required in hypoalbuminemic patients to achieve adequate diuresis. This finding is consistent with Lee et al. [18], who reported that the albumin–furosemide combination increased sodium excretion and urine output compared to furosemide alone, particularly in patients with low albumin levels. This combination appears beneficial for managing fluid overload and edema in diuretic-resistant patients [17,36].

Finally, the relationship between albumin levels and mortality in our study echoes the findings of Kempny et al. [37] highlighting hypoalbuminemia as a significant risk factor for mortality in CHD patients. Our data showed that hypoalbuminemia on the first and second postoperative days significantly increased mortality risk, with oliguric urine output rates further elevating this risk.

A statistically significant and positive relationship was detected between the albumin level on the first postoperative day and the urine output in the first 6 and 24 h of the postoperative period (*p* = 0.024 and *p* = 0.012). The relationship between furosemide and urine output rates in the postoperative period was positive and statistically significant. Regression analysis demonstrated that postoperative albumin levels positively affected furosemide efficacy and urine output within the first 24 h.

In our study, we observed that albumin levels on postoperative day 1 correlated with urine output within the first 6 and 24 h following surgery, indicating a potential early interaction between albumin concentration and furosemide efficacy. This relationship may be due to furosemide’s dependence on albumin for binding and transport to the kidneys, where it exerts its diuretic effect. In cases of hypoalbuminemia, furosemide’s binding is reduced, potentially limiting its availability at the target site and thus requiring higher doses to achieve similar diuretic responses.

Our findings suggest that maintaining adequate albumin levels in the immediate postoperative period could enhance furosemide’s efficacy, especially within the first 24 h. This may be particularly relevant for infants with congenital heart disease, where fluid balance is critical. The observed need for increased furosemide doses in hypoalbuminemic patients aligns with this temporal association, as the reduced binding efficiency in low-albumin states appears to impact furosemide availability and diuresis early on. Therefore, early postoperative albumin management may play a crucial role in optimizing diuretic therapy and improving patient outcomes.

Our study underscores the critical role of albumin levels and urine output in the postoperative management of pediatric patients undergoing cardiac surgery for congenital heart disease. These findings highlight the importance of preoperative and early postoperative albumin level monitoring as predictive markers for postoperative complications and outcomes. Hypoalbuminemia significantly correlates with increased mortality and morbidity, necessitating careful management to improve patient outcomes.

## 5. Conclusions

Our study underscores the critical role of albumin levels and urine output in the postoperative management of pediatric patients undergoing cardiac surgery for congenital heart disease. These findings highlight the importance of preoperative and early postoperative albumin level monitoring as predictive markers for postoperative complications and outcomes. Hypoalbuminemia significantly correlates with increased mortality and morbidity, necessitating careful management to improve patient outcomes.

This study also reinforces the efficacy of combining albumin with furosemide to enhance diuretic response, particularly in hypoalbuminemic patients. This approach can effectively manage fluid overload and reduce the risk of complications such as AKI and prolonged ICU stays. Monitoring urine output provides a practical and immediate assessment of drug response, aiding in timely interventions.

## Figures and Tables

**Table 1:** Demographical and clinical characteristics.

Parameters	Frequency
Male, *n* (%)	79 (42.5)
Female, *n* (%)	107 (57.5)
Age, median (min–max)	97.5 (1–360)
Body weight, kg, median (min–max)	4.3 (1.2–11)
Cardiopulmonary bypass duration, min, median (min–max)	138 (21–341)
Aortic clamp duration, min, median (min–max)	93.5 (8–312)
Postoperative pulmonary hypertension, *n* (%)	68 (36.5)
Peritoneum dialysis, *n* (%)	21 (11.2)
Sepsis, *n* (%)	32 (17.2)
Extracorporeal membrane oxygenation, *n* (%)	2 (1)
Mortality, *n* (%)	32 (17.2)
Duration in hospital, day, mean ± SD	29.85 ± 23.38

**Table 2:** Distribution of CHD in patients.

Types of CHD	Frequency
Atrial septal defect + ventricular septal defect *n* (%)	53 (28.5)
Hypoplastic left heart syndrome, *n* (%)	18 (9.6)
Complete atrioventricular canal defect, *n* (%)	16 (8.6)
Pulmonary atresia, *n* (%)	13 (6.9)
Fallot tetralogy, *n* (%)	11 (5.9)
Coarctation of the aorta, *n* (%)	7 (3.7)
Total anomalous pulmonary venous return, *n* (%)	7 (3.7)
Tricuspid atresia, *n* (%)	7 (3.7)
Others, *n* (%)	7 (3.7)
Transposition of Great Arteries, *n* (%)	4 (2.4)
Truncus arteriosus, *n* (%)	1 (0.5)

**Table 3:** Postoperative urine output categories and number of patients.

Patient (*n*)	Oliguria	Normal Urea	Polyurea
First 6 h (*n* = 162)	3 (%1.85)	72 (%44.4)	87 (%53.7)
First 24 h (*n* = 158)	2 (%1.27)	58 (36.71)	98 (62.03)
24–48 h (*n* = 152)		62 (%40.79)	90 (%59.21)
48–72 h (*n* = 132)		34 (%25.8)	98 (%74.2)

**Table 4:** Relationship between albumin therapy and urine output.

Urine Output (cc/kg/h)	No Albumin Treatment (*n* = 92)	With Albumin Treatment (*n* = 50)	*p*
Postoperative 6th h *	5.6 ± 3.4	4.4 ± 3.9	0.077
Postoperative 24th h *	5.6 ± 2.2	5.1 ± 3.1	0.299
Postoperative 24–48th h *	4.9 ± 2.1	5.99 ± 2.3	0.02
Postoperative 48–72nd h *	5.9 ± 2	6.2 ± 2.4	0.073

* mean ± SD.

**Table 5:** Correlations between albumin, furosemide, and urine output.

Variable		Furosemide	Urine Output			
			First 6 h	First 24 h	24–48 h	48–72 h
Albumin Day 1	r	−0.098	0.179	0.201	−0.031	0.013
	p	0.208	0.024	0.012	0.709	0.886
	n	166	159	157	150	131
Albumin Day 2	r	0.219			−0.099	−0.165
	p	0.008			0.247	0.070
	n	148			139	121
Albumin Day 3	r	0.001				0.044
	p	0.989				0.658
	n	122				104
Furosemide	r		0.328	0.076	0.211	0.185
	p		<0.001	0.036	0.011	0.036
	n		148	148	144	128

**Table 6:** Effect of albumin on furosemide and urine output.

	β ± Standard Error	*p*	%95 Confidence Level Lower-Upper Bound
Constant	−33.58 ± 4.89	<0.001	(−43.312)–(−23.848)
Furosemide	4.32 ± 0.94	<0.001	2.453–6.192
Urine output (First 24 h)	2.24 ± 0.73	0.003	0.776–3.708

**Table 7:** Effect of postoperative albumin levels and urine output on mortality.

Postoperative	β ± Standard Error	Odds	%95 Confidence Level Lower-Upper Bound	*p*
Albumin day 1 (Hypoalbuminemia)	1.95 ± 0.42	7.03	3.09–15.99	<0.001
Albumin day 2 (Hypoalbuminemia)	1.35 ± 0.51	3.84	1.41–10.44	0.008
Albumin day 3 (Hypoalbuminemia)	0.84 ± 0.54	2.31	0.80–6.66	0.120
Urine output, first 6 h (Oliguria)	−0.44 ± 0.14	0.64	0.49–0.83	0.001
Urine output, first 24 h (Oliguria)	−0.62 ± 0.17	0.54	0.39–0.75	<0.001
Urine output, 24–48th h (Oliguria)	0.13 ± 0.11	1.14	0.92–1.40	0.241
Urine output, 48–72nd h (Oliguria)	0.11 ± 0.14	1.11	0.84–1.47	0.450

**Table 8:** Ejection fraction (EF) values in hypoalbuminemic and normoalbuminemic groups.

Parameter	Hypoalbuminemic (*n* = 36)	Normoalbuminemic (*n* = 150)	*p*-Value
EF (%), mean ± SD	55 ± 10	60 ± 8	<0.05

EF: ejection fraction, SD: standard deviation.

## Data Availability

Datasets analyzed or generated during this study are presented in the current study.
